# Targeting the hotspots: investigating spatial and demographic variations in HIV infection in small communities in South Africa

**DOI:** 10.1186/1758-2652-13-41

**Published:** 2010-10-27

**Authors:** Handan Wand, Gita Ramjee

**Affiliations:** 1 National Centre in HIV Epidemiology and Clinical Research, Sydney, Australia; 2 HIV Prevention Research Unit, Medical Research Council, Durban, South Africa

## Abstract

**Background:**

In South Africa, the severity of the HIV/AIDS epidemic varies according to geographical location; hence, localized monitoring of the epidemic would enable more effective prevention strategies. Our objectives were to assess the core areas of HIV infection in KwaZulu-Natal, South Africa, using epidemiological data among sexually active women from localized communities.

**Methods:**

A total of 5753 women from urban, peri-rural and rural communities in KwaZulu-Natal were screened from 2002 to 2005. Each participant was geocoded using a global information system, based on residence at time of screening. The Spatial Scan Statistics programme was used to identify areas with disproportionate excesses in HIV prevalence and incidence.

**Results:**

This study identified three hotspots with excessively high HIV prevalence rates of 56%, 51% and 39%. A total of 458 sexually active women (19% of all cases) were included in these hotspots, and had been exclusively recruited by the Botha's Hill (west of Durban) and Umkomaas (south of Durban) clinic sites. Most of these women were Christian and Zulu-speaking. They were also less likely to be married than women outside these areas (12% vs. 16%, p = 0.001) and more likely to have sex more than three times a week (27% vs. 20%, p < 0.001) and to have had more than three sexual partners (55% vs. 45%, p < 0.001). Diagnosis of genital herpes simplex virus type 2 was also more common in the hotspots. This study also identified areas of high HIV incidence, which were broadly consistent with those with high prevalence rates.

**Conclusions:**

Geographic excesses of HIV infections at rates among the highest in the world were detected in certain rural communities of Durban, South Africa. The results reinforce the inference that risk of HIV infection is associated with definable geographical areas. Localized monitoring of the epidemic is therefore essential for more effective prevention strategies - and particularly urgent in a region such as KwaZulu-Natal, where the epidemic is particularly rampant.

## Background

It is estimated that more than 60% of the world's HIV-infected population lives in sub-Saharan Africa, and South Africa is currently experiencing the heaviest HIV/AIDS load in the world [1]. In South Africa's province of KwaZulu-Natal, the epidemic is at the most advanced stage, with HIV prevalence among mothers attending antenatal clinics estimated to be 39% [2]. Reasons as to why the HIV epidemic is rampant in this region are likely to be multi-factorial and complicated. Socio-economic conditions and specific factors, such as patterns of sexual networking, levels of condom use and sexually transmitted infections, are known to be important determinants of spread of HIV infection [2,3].

Use of current HIV prevention methods, such as condoms, monogamy and abstinence, is not always realistic in practice for many reasons. The need for improved preventative technologies against HIV infection remains urgent. Researchers are trying to develop an effective microbicide that could be used by women to help prevent HIV transmission. However, clinical trials of the efficacy of microbicides have so far proved disappointing [4-7].

As the epidemic continues its devastating impact in this region, geostatistical approaches have received increasing attention as a way of determining possible "hotspots" of HIV infection and prioritizing areas for intervention [8,9]. If found to exist and to have significantly excessive rates of HIV, such hotspots could be considered as surrogates for unobserved or unknown risk factors.

However, investigating the spatial structure of the HIV epidemic can be challenging. Sparsely populated, large geographical areas can mask geographical heterogeneity and may potentially cause misinterpretation of true underlying geographical patterns [10].

The HIV Prevention Research Unit of the Medical Research Council in Durban, KwaZulu-Natal, has been involved in many international research programmes and clinical trials in HIV prevention, playing an important role in the fight against HIV (G Ramjee, personal communication). The role of this unit includes teaching thousands of women about caring for themselves, including using condoms, and encouraging them to test for HIV, as well as helping those already infected.

In this study, we investigated the geographical clustering of HIV infection using data from six geographical strata; these came from two of the unit's site-preparedness studies and one HIV prevention phase III clinical trial of vaginal diaphragms. The cohorts of women were drawn from rural, semi-rural and urban communities in KwaZulu-Natal.

The geographical data (latitude and longitude) obtained using the geographic information system (GIS) and global positioning system (GPS) technologies were fed into a statistical programme [10] to characterize spatial clusters of HIV infections without previous knowledge of either the number or location of the clusters [11]. A "cluster" or "hotspot" is detected within a defined geographical location during a specific timeframe when the location has a disproportionate excess of HIV infections when compared with neighbouring areas under study.

We hypothesized that geographical clusters of HIV infection would represent location-specific networks, and could be used as the basis to link socio-demographic data to show that these clusters represent relatively homogenous groups of women, thus allowing a large sexual network to be divided into smaller sub-communities. We also addressed the question of whether or not other demographic or sexual behavioural data could further differentiate the geographically distinct clusters in this region. Such data could provide valuable insight into the spread of HIV infection.

## Methods

### Study areas and geographical data

We combined data from 5753 sexually active women who consented to screening for three studies from six clinics and 158 census locations included in this study. The studies were as follows: the Methods for Improving Reproductive Health in Africa (MIRA) trial of the diaphragm for HIV prevention, September 2002 to September 2005 (rural Umkomaas, 44 km south of Durban, and Botha's Hill, 31 km west of Durban) [12]; the Microbicides Development Programme (MDP) Feasibility Study in Preparation for Phase III Microbicide Trials, August 2002 to September 2004 (semi-rural Tongaat, 31 km north of Durban, and Verulam, 22 km north of Durban) [13]; and the HIV Prevention Trials Network (HPTN 055) Site-preparedness Study for Future Implementation of PhaseII/IIb/III clinical trials, May 2003 to January 2005 (rural district of Hlabisa and urban Durban) [14].

Details of participants' places of residence were collected on a locator information form at screening, and residential areas were captured onto a spreadsheet. Field staff visited each participant's place of residence; once an appropriate satellite fix was acquired, the coordinates were recorded on a hand-held GPS device, and a back-up hard copy of the data was also created.

Participants' confidentiality was ensured by using identifying numbers linked to the GPS coordinate reading, instead of names and addresses. At the end of each working day, field staff captured the coordinates digitally on a spreadsheet. These data were forwarded to the GIS lab and geographical coordinates for each of 158 census locations were used as a proxy for the location of participants in the study.

For the MIRA and HPTN 055 trials, HIV diagnostic testing was achieved using two rapid tests on whole blood sourced from either finger-prick or venepuncture (Determine HIV-1/2, Abbot Laboratories, Tokyo, Japan and Oraquick, Orasure Technologies, Bethlehem, PA, USA). During the MDP feasibility study, the Abbot IMX ELISA test (Abbot Diagnostics, Africa Division), in combination with the Vironostika HIV1/2 ELISA for positive and equivocal results, was used on whole blood sourced from venepuncture. Only women who had a test result and geographical data were included in the study.

The main eligibility criteria were consistent across the trials and included: being sexually active; being HIV negative at screening at inclusion; willingness to provide written consent and follow study procedure; not being pregnant and with intention to maintain this status; and residing in and around the study area for a minimum of one year. At all visits, all participants received counselling on risk reduction and as many male condoms as desired. Counsellors emphasized that condoms are the only known method to prevent HIV and sexually transmitted infections (STIs), and that condoms should be used for every act of sex.

Women who were identified as HIV positive at screening were referred to local health care facilities for care and support. Women who seroconverted during the trial remained in the study and were provided with ongoing counselling and referred to local health care facilities for further care at the end of the study. The protocol and informed consent forms were approved by the respective ethics committees at each site.

### Spatial scan methodology

The geographical data obtained from GIS/GPS techniques were used to determine the potential areas with an excess of HIV infection by using the Spatial Scan Statistics (SaTScan) programme developed by Kulldorf [15]. This has become the most widely used test for clustering in recent years, both because of its efficacy in detecting single "hotspots", as well as availability of the free software package [16] for implementing the test. The basic idea is to allow circular windows of various sizes to range across the study region; at each location, the rate of disease inside the window is compared with that outside of it.

A Poisson-based model was chosen, where the number of HIV counts in an area is Poisson distributed according to a known underlying population at risk. Under the Poisson assumption, the likelihood function for a specific window is proportional to:

$$\left(\frac{c}{E[c]}\right){\left(\frac{C-c}{C-E[c]}\right)}^{C-c}I$$

where *C *is the total number of cases, *c *is the observed number of cases within the window, and *E*[*c*] is the covariate adjusted expected number of cases within the window under the null hypothesis. Since the analysis is conditioned on the total number of cases observed, *C- E*[*c*] is the expected number of cases outside the window. *I *is an indicator function. When SaTScan is set to scan only for clusters with high rates, *I *is equal to 1 when the window has more cases than expected under the null-hypothesis, and 0 otherwise.

For a given zone (circular window), the methodology calculates the probability of a data point being a case inside or outside the circle under consideration. For each circle, a likelihood ratio is computed for the alternative hypothesis that there is an increased risk of disease inside the circle, against the null hypothesis that the risk inside the circle is the same as that outside. In this context, a cluster or hotspot is said to be detected within a defined geographical area during a specific timeframe if the area has a disproportionate excess of HIV cases when compared with neighbouring areas under study.

By meeting the statistical assumptions of a set of statistical models, an unusual rise or reduction in cases in a specific spatial area can be characterized by statistical significance. The sets of potential clusters are then rank-ordered according to the magnitude of their likelihood ratio test statistics.

Once the null hypothesis is rejected and clusters are formed, this means that the number of HIV infections detected in this region is significantly different from those in other study areas. Socio-demographic and behavioural characteristics of the women within these hotspots were compared with those of women outside of them. Cluster detection analysis was restricted to the "spatial option" only because the temporal variation in this study was not large enough to detect any temporal clusters.

The user-defined maximum radius used by SaTScan was set to its default value of 50%, as recommended by Kulldorf [17] as optimal. In order to investigate the sensitivity of SaTScan results to the default setting, we ran the SaTScan spatial scan statistics 10 times, starting with a maximum size of 5% and increasing the parameter by an interval of 5% with each run until reaching the default maximum size value of 50%. Results were not affected by the choice of radius selected; we therefore used the default value of 50% in our analysis.

The Chi-square test was used to compare differences in proportions, and Student's *t *test (a nonparametric test) to compare differences in continuous variables.

Calculations were carried out using SaTScan version 8.0 http://www.satscan.org, and results were imported into the Stata (Version 10.0, CS, TX) software environment to compare the characteristics of cluster (hotspots) and non-cluster areas.

## Results

**Figure 1:**
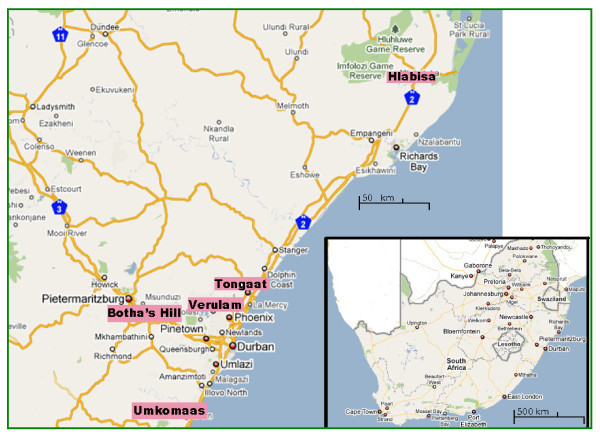
**Study locations**.

As described earlier, our study included women who consented to participate in one of three studies from six clinic sites and 158 census locations, from among a total black female population of ~2,400,000. Figure 1 presents the location of the study areas. The geographical data of a total of 2369 women who tested positive for HIV infection at a follow-up screening were used to determine high HIV prevalence areas. Added to this were 211 women who were HIV negative at screening but who seroconverted during follow up.

### Hotspots of increased HIV prevalence

Table 1 shows the results from the SaTScan tests for significant spatial clustering in terms of HIV prevalence, after adjusting for size of the underlying population at risk and for age.

**Table 1:** SaTScan test results for significant spatial clustering in terms of HIV prevalence among sexually active women after adjusting for size of the underlying population at risk and for age.

Potential clusters*	Radius (km)	Prevalence of HIV (%)	Total women tested	Relative risk of excess HIV cases	p-values
Cluster No. 1	4.5	56.0	315	34.6	0.001
Cluster No. 2	32.0	51.0	569	2.4	0.001
Cluster No. 3	3.7	39.0	59	10.1	0.001

*1 - Inchanga, Hammersdale (west of Durban); 2 - Mthwalume, Umzinto, Molweni (south of Durban); 3 - Hillcrest, Botha's Hill (west of Durban).

**Figure 2:**
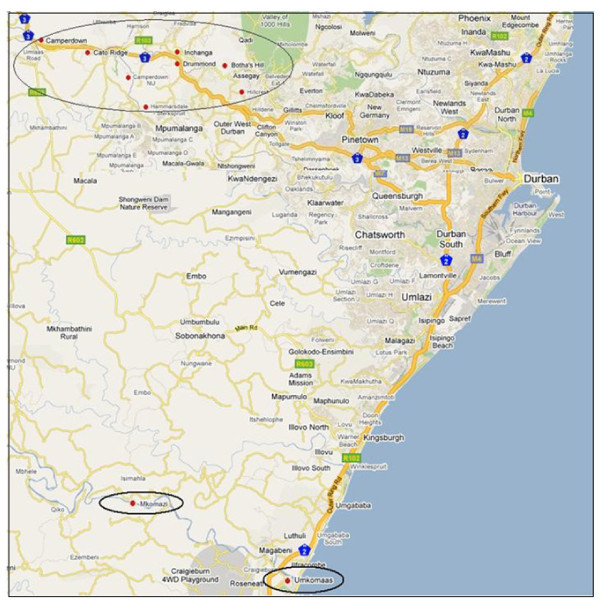
**Geographical locations of clusters (high prevalence and high incidence of HIV)**. Inchanga and Hammersdale: High prevalence and high incidence (Durban West). Hillcrest and Botha's Hill: High prevalence and high incidence (Durban West). Camperdown and Cato-Ridge: High incidence (Durban West). Umkomaas and Mkomanzi: high incidence (Durban South).

Analysis identified three hotspots or clusters of prevalence, and these included 458 cases (19% of all) recruited at two study sites: a less urbanized clinic in Botha's Hill and a peri-urban clinic in Umkomaas. These three hotspots were determined to be areas of particularly high prevalence when compared with other study sites (Verulam, Tongaat, Hlabisa and Durban) (Figure 2).

In one cluster, 144 (31%) HIV cases were determined to be centred within a 4.5 km radius in Inchanga and Hammersdale (relative risk [RR] = 34.70, p = 0.001), west of Durban. The second cluster included 168 (37%) HIV cases within a 32 km radius in the south of Durban, from the three residential areas of Umzinto, Molweni and Mtwalume (RR = 2.4, p = 0.001). Like the first, the third cluster was again located west of Durban, with 146 (32%) HIV cases (RR = 10.1, p = 0.001) in residential areas encompassing Hillcrest and Botha's Hill.

Distribution of the demographic characteristics and reported sexual behaviour of women who fell within the cluster areas or hotspots were compared with those who did not (Table 2).

**Table 2:** Characteristics of sexually active women who fell within the hotspots compared with those who did not

Screening characteristics	Inside the clusters	Outside the clusters	P value
HIV positive	49%	39%	< 0.001

Age (yrs)			0.548
≤ 24	40%	42%	
25-34	38%	36%	
35+	22%	22%	
Less than high school education	27%	26%	0.481
Married	12%	16%	0.001
Living with a regular partner	29%	31%	0.301
Language of screening form			
English	8%	13%	< 0.001
Zulu	91%	86%	
Other	1%	1%	
Religion (Christian)	94%	90%	< 0.001
Lifetime number of sexual partners			< 0.001
1	20%	26%	
2	25%	29%	
3+	55%	45%	
Age at first sex < 17 yrs	87%	86%	0.270
Coital frequency (≥ 3 times/week)	27%	20%	< 0.001
Tested positive for STIs	16%	16%	0.987
Chlamydia	9%	9%	0.600
Gonorrhea	3%	3%	0.853
*Trichomonas vaginalis*	6%	6%	0.864
Tested positive for HSV2 at screening	77%	71%	< 0.001
Current contraceptive use at screening^1^	78%	78%	0.835

Information on condom use was not available at the screening.

^1^Any of the following: long term (vasectomy, tubal ligation, "Jadel", "Norplant", "Noplant", "removed uterus"), injectable hormones, the pill, barrier (male/female condoms) and other/none.

Women who fell within one of the three hotspots were similar in terms of age (p = 0.548) and education level (p = 0.481) to those who did not. Proportions of women were similar between those in the hotspots and those who were not in terms of those living with a regular sex partner (p = 0.301], age at first sex < 17 years (p = 0.270), being diagnosed with an STI (chlamydia, gonorrhoea, syphilis or *Trichomonas vaginalis*) (p = 0.987) and current contraceptive use (p = 0.835).

The proportion of women who reported being legally married was significantly higher among those outside the hotspots than within them (16% vs. 12%, p = 0.001). Significantly more women in the geographical hotspots reported being Christian (94% vs. 90%, p < 0.001) and speaking Zulu at home (91% vs. 86%, p < 0.001) compared with those in non-cluster areas.

More women within the hotspots reported having sex an average of three or more times per week (27% vs. 20%, p < 0.001) and to having three or more sexual partners in their lifetime (55% vs. 45%, p < 0.001) compared with those outside the hotspots. Also, significantly more women within the hotspots were diagnosed with genital herpes simplex virus type 2 (HSV-2) than those not in these areas (77% vs. 71%, p < 0.001).

### Hotspots of HIV incidence

A total of 2523 HIV-positive women enrolled in the three studies were eligible, with a median duration of follow up of 12 months. Of these, 211 had seroconverted during the follow-up period (incidence rate 6.6/100 women-years). Using the SaTScan programme, and adjusting for the underlying population at risk and age, a total of 48 of the women who seroconverted (22% of all HIV seroconversions) were geographically clustered into four hotspots (Table 3). Two of these clusters overlapped with the high HIV prevalence hotspots located west of Durban.

**Table 3:** Distribution of cases of HIV seroconversion during follow up that fell into four clusters (n = 48)

Potential clusters*	Radius (km)	Total women tested	Relative risk of excess HIV cases	p-values	Total locations
Cluster No. 1	4.5	137	22.1	0.001	2
Cluster No. 2	4.3	31	19.4	0.001	2
Cluster No. 3	2.6	260	11.8	0.001	2
Cluster No. 4	3.7	26	9.2	0.001	2

1 - Inchanga, Hammersdale (west of Durban); 2 - Camperdown, Cato Ridge (west of Durban); 3 - Umkomaas, Mkomanzi (south of Durban);

4 - Hillcrest, Botha's Hill (west of Durban).

The highest incidence of HIV infection was observed in a hotspot that comprised two census areas west of Durban, namely Inchanga and Hammersdale, encompassing a radius of 4.5 km (RR = 22.1, p < 0.001). The second hotspot included Camperdown and Cato Ridge (RR = 19.4, p < 0.001) and another included Hillcrest and Botha's Hill (RR = 9.2, p < 0.001), both located west of Durban, within 4.3 km and 3.73 km radii, respectively. The fourth hotspot included Umkomaas and Mkomanzi (RR = 11.8, p < 0.001) south of Durban.

## Discussion

Our study identified three localized hotspots of high HIV prevalence; two of these were exclusively located west of Durban and included women from two of the clinical sites. In addition, four hotspots of high HIV incidence were found, two of which overlapped with high HIV prevalence areas and also comprised census areas west of Durban.

The Spatial Scan Statistics programme was used to investigate geographical patterns and variations in HIV prevalence within the relatively homogeneous population. Strong statistical evidence of clustering of HIV infections in communities of Durban was found. This supports the notion that risk factors for HIV might be associated with certain specific socio-economic characteristics, which could be targeted to improve existing public health prevention measures aimed at the general population.

Prevalence of HIV infection in South Africa has always been reported either on a national basis or as a provincial average [2]. While it is necessary and important to report these figures at national level, such aggregate estimates may mask the spatial heterogeneity of the HIV epidemic. Hence, national level prevalence rates may not reveal the full impact of the epidemic on different geographical regions. It is evident, as this study indicates, that the epidemic should be monitored in a localized way so that more effective prevention strategies may be utilized. This is particularly urgent and necessary in a region such as KwaZulu-Natal, where the epidemic continues its rampant pace with devastating impact.

The results from this study support the conclusion that risks for HIV infection are associated with definable socio-demographic factors, which may be fundamental ecological units of HIV transmission [10]. A multitude of other factors may have an impact in these mostly rural or peri-rural settings, creating a context in which the impact of geographical factors and sexual behaviours on HIV prevalence and incidence may be particularly relevant.

The spatial clustering of HIV cases was found to be related to certain demographic and risk behaviours. Number of male sexual partners was not collected in this study; however, being single, combined with high frequency of sexual acts, gives strong evidence for those women having multiple partners, as well as possibly engaging in transactional sex.

These results may be due to fundamental differences between the communities with regard to health care centres, population density and other socio-economic factors. These data provide new evidence to support the need to investigate potential sources of infection and to study transmission patterns in the community in order to apply relevant interventions for prevention of this devastating disease.

Our data suggest strategies for targeted control and for prioritization of scarce resources. A community-based prevention programme could be formulated to educate residents in these endemic areas about the risks associated with HIV and other high-risk sexual behaviours.

Information on the spatial distribution of populations and services is essential to understand access to health services. There should be specially focused strategies to optimize health care for people living in the high-risk areas. Spatial analysis is an important tool for monitoring the HIV epidemic, predicting future treatment demands, and targeting areas for public health interventions. The mapping of areas of high HIV prevalence will aid community interventions, such as education, prevention, treatment and care, and optimum location of referral health centres.

The strength of our study is that we were able to use data from a region that is at the epicentre of the HIV epidemic in South Africa, if not the world, to determine core areas of the epidemic.

Our study has some limitations that need to be considered in the interpretation of the results. First, because of the nature of the research conducted in these trials, populations selected were known to be moderate-to-high risk of HIV infection. Although we were able to target women from different communities in different settings (rural, semi-rural and urban), the women in this study may not necessarily be representative of women in the KwaZulu-Natal province. Second, this analysis is that sexual networks may be subject to temporal trends, which we were not able to determine. Third, we were unable to collect any sexual behaviour data from male partners of the women, which can have a substantial impact on the results. Therefore, additional research is required to fully understand the reasons for these spatial variations in HIV infections in this region, and important insights will be gained by further in-depth study of the communities identified in this study.

Another limitation of the approach used in the present study is the circular nature of the SaTScan window; SaTScan identifies clusters by imposing circular windows on maps and allowing the size of these to vary between zero and a preset upper limit. Although this may work well on maps that show relatively large geographical units (such as those used in the present study), it may not work as well on a smaller scale, where neighbourhood-level geographical barriers, such as rivers or train tracks, could create non-circular interaction patterns. However, the Spatial Scan Statistics employed in the present study has higher statistical power than other geostatistical methods and has been widely applied to the detection of clustering of diseases [18-21].

## Conclusions

We investigated spatial and demographic variations in HIV infection in small communities in KwaZulu-Natal, South Africa, making use of a cohort of women recruited for various trials through population-based clinics. HIV prevalence rates have always been higher in KwaZulu-Natal than in any other province in South Africa, and this trend has been sustained since the early 1990s. Our findings are consistent with previous work in this population [2,3,9]. However, our results also showed considerable variation within the province of KwaZulu-Natal, which cannot be detected in an aggregated data.

An understanding of geographical variation and determination of the core areas of the disease may provide an explanation regarding possible proximal and distal contributors to the HIV/AIDS epidemic. It is more urgent than ever to determine and target the specific communities that are most in need of education, prevention and treatment activities.

This study provides a first attempt to visually and quantitatively describe the geographical characteristics of HIV infections in a region where the disease is known to be rampant. The results may inform development of prevention programmes to address the HIV epidemic while considering those groups most affected differentially by geographical area.

Investigating the geographical structure of the HIV epidemic in sparsely populated, large geographical areas is challenging, if not impossible. There needs to be urgent public demand for monitoring at localized level, designating the resources carefully to those places where the infection is clustered. We provide evidence of clusters of particularly vulnerable women through research on the prevalence and incidence of HIV in our setting, and would urge the authorities to provide a rapid response by scaling up HIV prevention, treatment and care efforts in all these communities.

## Competing interests

The authors declare that they have no competing interests.

## Authors' contributions

Both authors contributed to the manuscript, and saw and approved the final version. HW carried out the analyses and drafted the manuscript. GT participated in the design of the study and drafted the manuscript. Both authors read and approved the final manuscript.
